# Multiple Roles of Angiopoietin-Like 4 in Osteolytic Disease

**DOI:** 10.3389/fendo.2017.00080

**Published:** 2017-04-18

**Authors:** Helen J. Knowles

**Affiliations:** ^1^Nuffield Department of Orthopaedics Rheumatology and Musculoskeletal Sciences, University of Oxford, Oxford, UK

**Keywords:** angiopoietin-like 4, bone resorption, cartilage degradation, angiogenesis, inflammation, rheumatoid arthritis

## Abstract

Hypoxia and the hypoxia-inducible factor (HIF) transcription factor drive pathological bone loss in conditions including rheumatoid arthritis (RA), osteoarthritis, osteoporosis, primary bone tumours, and bone metastatic cancer. There is therefore considerable interest in determining the function(s) of HIF-induced genes in these pathologies. Angiopoietin-like 4 (ANGPTL4) is an adipose-derived, HIF-1α- and PPARγ-induced gene that was originally discovered as an endocrine and autocrine/paracrine regulator of lipid metabolism. Given the inverse relationship between bone adiposity and fracture risk, ANGPTL4 might be considered a good candidate for mediating the downstream effects of HIF-1α relevant to osteolytic disease. This review will consider the possible roles of ANGPTL4 in regulation of osteoclast-mediated bone resorption, cartilage degradation, angiogenesis, and inflammation, focusing on results obtained in the study of RA. Possible roles in other musculoskeletal pathologies will also be discussed. This will highlight ANGPTL4 as a regulator of multiple disease processes, which could represent a novel therapeutic target in osteolytic musculoskeletal disease.

## Introduction

Bone remodelling is a carefully regulated process that requires the coordinated actions of osteoclasts, which resorb bone, and osteoblasts, which form new mineralised bone. The remodelling process is essential for formation, development, and maintenance of the skeleton. Disruption of the balance between bone formation and bone resorption in favour of osteoclast overactivation results in pathological bone loss, as evident in osteolytic conditions including rheumatoid arthritis (RA) (1, 2), osteoporosis (3), primary bone tumours (4–6), and bone metastatic cancer (7). The same conditions are associated with microenvironmental hypoxia, which correlates with disease progression and reduced chance of survival (8–11).

Hypoxia-inducible factor (HIF) is a critical mediator of cellular responses to hypoxia. HIF is a heterodimeric transcription factor that is regulated at the level of the stability and transcriptional activity of the alpha subunits (HIF-1α, HIF-2α). In normoxic conditions, HIF-α is posttranslationally hydroxylated by the prolyl hydroxylase domain enzymes (PHD1–3), which target it for proteasomal degradation, and asparagine hydroxylase factor-inhibiting HIF (FIH), which inhibits any remaining transcriptional activity. However these enzymes are oxygen dependent, allowing HIF-α to stabilise under hypoxia and bind to the hypoxia-response element of HIF target genes to initiate hypoxia-induced transcription (12).

As hypoxia and HIF drive disease progression in various musculoskeletal conditions, there is considerable interest in the pathological function(s) of HIF-induced genes. Angiopoietin-like 4 (ANGPTL4) is a secreted adipokine and a member of a family of eight angiopoietin-like (ANGPTL1–8) proteins. Hypoxic induction of ANGPTL4 by the HIF-1α isoform of HIF was initially described in cardiomyocytes (13) but also occurs in other musculoskeletal cells including adipocytes (14), endothelial cells (15), chondrocytes (16), monocytes, osteoclasts, and osteoblasts (17).

Despite being structurally similar to the angiopoietins, ANGPTLs do not bind either the Tie1 or Tie2 receptor and have no identified cognate receptors, rendering them orphan ligands. Full-length ANGPTL4 (flANGPTL4) contains a signal peptide mediating its secretion, an N-terminal coiled-coil domain, a linker, and a C-terminal fibrinogen-like domain (18). This 406 amino acid glycosylated protein, with a molecular mass of approximately 65 kDa, can be proteolytically cleaved at the linker region by proprotein convertases to generate N-terminal (nANGPTL4) and C-terminal (cANGPTL4) fragments (19). Both flANGPTL4 and nANGPTL4 oligomerise *in vivo*, whereas cANGPTL4 dissociates into monomers (20, 21). Cleavage of ANGPTL4 appears to be tissue dependent; the human liver secretes cleaved ANGPTL4, whereas adipocytes secrete the full-length form (22, 23). The three forms of ANGPTL4 exert distinct physiological functions; regulation of lipid metabolism is the primary function of N-terminal ANGPTL4 (20, 22).

Angiopoietin-like 4 was initially discovered as a central regulator of lipid metabolism that was induced by PPARγ under fasting conditions, accounting for its initial nomenclature of fasting-induced adipose factor (FIAF) (24). It is also transcriptionally regulated by PPARα and β/δ (22, 25). ANGPTL4 is the primary physiological regulator of lipoprotein lipase (LPL) activity, stimulating conversion of catalytically active LPL dimers into inactive monomers. This causes increased levels of plasma triacylglycerol, specifically VLDL, and non-esterified fatty acids, with subsequent depletion of adipose tissue stores (26, 27).

The relationship between fat and bone is complex. Body weight positively associates with bone mineral density, but bone marrow adiposity and bone mass exhibit an inverse relationship, and many conditions associated with increased fracture risk display increased marrow adiposity (28, 29). As an adipose-derived factor, it seems likely that ANGPTL4 might also play physiological and pathological roles within the skeleton. This review will consider possible roles for ANGPTL4 in osteolytic disease, particularly focussing on the pathogenesis of RA.

## Pathological Functions of ANGPTL4 in RA

Rheumatoid arthritis is a chronic inflammatory disease characterised by the formation of a hyperplastic synovium containing synovial fibroblasts, macrophages, CD4+ T cells, B cells, and plasma cells. The synovium is locally invasive and, alongside activated osteoclasts, erodes articular cartilage and subchondral bone causing progressive destruction of the affected joints, associated with joint pain and compromised function. Synovial hyperplasia increases the distance between synovial lining cells and the nearest blood vessel. This ultimately exceeds the diffusion limit for oxygen, resulting in the development of a hypoxic microenvironment within the RA synovium that correlates with the intensity of inflammation and is a poor prognostic indicator (11). Both HIF-1α and HIF-2α are overexpressed in RA, and the HIF pathway is considered a target for therapy (30).

Angiopoietin-like 4 overexpression was first described in stromal fibroblasts within the joints of mice with collagen-induced arthritis (31). It has since been reported in RA articular chondrocytes (16) and in stromal fibroblasts, macrophages, plasma cells, endothelial cells, and osteoclasts within the hyperplastic synovium (32). Such widespread induction could rapidly provide a large local pool of ANGPTL4 to regulate a variety of disease processes. ANGPTL4 was also elevated in the serum and synovial fluid of RA patients in comparison with non-inflammatory osteoarthritis (OA) or normal controls (32).

### Osteoclast-Mediated Bone Resorption

Osteoclasts are large multi-nucleated cells that form by fusion of CD14+ monocytic precursors in the presence of macrophage colony-stimulating factor (M-CSF) and receptor activator of nuclear factor kappa B ligand (RANKL). Osteoclast differentiation is stimulated by hypoxia–reoxygenation, rather than hypoxia *per se*, and is not apparently dependent on HIF (33). However, bone resorption by mature osteoclasts is induced by hypoxia in a HIF-1α-dependent manner *in vitro* (17, 34–36) and *in vivo* (10). Similarly, PPARγ promotes osteoclast differentiation and bone resorption (37).

We reported hypoxia-inducible, HIF-1α-dependent induction of ANGPTL4 by human osteoclasts, as well as monocytes and osteoblasts (17). Exposure of mature human osteoclasts to flANGPTL4 stimulated a twofold to threefold increase in lacunar bone resorption that was independent of RANKL, while not affecting osteoclast differentiation (17). In contrast, Lin et al. reported neither flANGPTL4 nor cANGPTL4 to affect either murine osteoclast formation or bone resorption, whereas nANGPTL4 inhibited both activities (38). Effects of nANGPTL4 were apparently mediated by reduced expression of RANKL, M-CSF, and connective tissue growth factor (CTGF) by stromal cells within the marrow culture, resulting in reduced expression of osteoclastogenic NFATc1 and DC-STAMP (38). The differences between the two studies may be due to interspecies effects or to the different osteoclast culture methods used.

It is currently unknown whether ANGPTL4 cleavage occurs in skeletal tissue, although adipocytes, which are numerous in the bone marrow, secrete the full-length protein (22, 23). We could detect only flANGPTL4 in human osteoclasts, but primary human osteoblasts produced both flANGPTL4 and cANGPTL4 *in vitro* (17). In support of a role for ANGPTL4 in osteoclast-mediated bone resorption, we correlated high serum concentrations of ANGPTL4 in RA with elevated levels of circulating RANKL, a serum marker of bone resorption (32).

### Cartilage Destruction

Articular cartilage is composed predominantly of chondrocytes and is avascular, meaning that the chondrocytes normally reside in a hypoxic environment. Destruction of articular cartilage in RA is associated with increased expression and activity of matrix metalloproteinases (MMPs). *In vitro* work on RA synovial fibroblasts describes hypoxia- and HIF-1α-driven induction of MMP expression by these cells (30), which could drive hypoxic enhancement of cartilage destruction.

Angiopoietin-like 4 might mediate a component of the hypoxic induction of MMPs. RA articular chondrocytes cultured *in vitro* exhibited hypoxia-inducible ANGPTL4 secretion. Similarly, normal human cartilage expressed little ANGPTL4 whereas strong cytoplasmic staining of articular chondrocytes occurred in more severely hypoxic rheumatoid cartilage (16). Exposure of articular chondrocytes to flANGPTL4 increased expression of MMP-1 and MMP-3 (16). cANGPTL4 also promoted cartilage matrix remodelling during chondrogenic differentiation, inhibiting aggrecan and type II collagen expression and inducing expression of MMP-1, MMP-3, and MMP-13 (39). ANGPTL4 might therefore contribute to cartilage matrix destruction in RA *via* induction of MMPs.

Angiopoietin-like 4 could also exacerbate cartilage destruction *via* promotion of osteoclast-mediated resorption pathways. Multi-nucleated cells resorbing cartilage in RA have an osteoclast-like phenotype, and human monocyte-derived osteoclasts can digest cartilage matrix *in vitro* (40, 41). As the erosive effect of osteoclasts on cartilage appear to be MMP-mediated (41), ANGPTL4 might induce cartilage erosion in RA, *via* effects on MMP production and osteoclast activation, to increase joint destruction.

### Angiogenesis

Synovial angiogenesis in RA probably occurs as a consequence of synovial hypoxia. Hypoxia-induced HIF induces expression of pro-angiogenic mediators including vascular endothelial growth factor (VEGF), interleukin 8 (IL-8), macrophage inflammatory protein 3α (MIP-3a), and stromal-derived factor 1 (SDF-1). The increased blood supply transports nutrients and immune cells to the inflammatory synovium but cannot provide sufficient oxygen to negate the hypoxic stimulus (30, 42). Increased ANGPTL4 expression during early stages of murine collagen-induced arthritis occurred specifically in stromal fibroblasts adjacent to blood vessels, suggestive of a role in angiogenesis (31).

Besides lipid regulation, the other main function of ANGPTL4 is vascular, with roles in angiogenesis and vessel permeability mediated by flANGPTL4 and cANGPTL4. ANGPTL4 is reported to inhibit endothelial apoptosis and stimulate endothelial cell migration and tube formation, so inducing angiogenesis *in vivo* (15, 18, 25, 31, 43–47). However, anti-angiogenic effects are also reported (48–52). The complexity of the response is highlighted by reports from the same group describing pro- and anti-angiogenic effects of ANGPTL4 (15, 49, 53) and by reports of opposing effects for both flANGPTL4 and cANGPTL4. There is similar controversy regarding whether ANGPTL4 promotes or inhibits vascular permeability (54, 55).

### Inflammation

Synovitis is a major characteristic of RA and HIF acts as a key regulator of the associated inflammation, being highly expressed in immune cells, especially macrophages, in the RA synovium (42, 56). Conditional knockout of HIF-1α in myeloid cells in a murine model of RA significantly reduced synovial inflammation and disease progression (57). Similarly, HIF-1α played a critical role in hypoxia-induced synovial hyperplasia and inflammatory cell infiltration in murine collagen-induced arthritis (58). HIF also induces expression of inflammatory cytokines including IL-6, IL-8, TNF-α, and IL-1β in rheumatoid synovial fibroblasts (59).

Angiopoietin-like 4 is also overexpressed in inflammation. ANGPTL4 was induced by IL-1β in osteoblasts (60) and by IL-1β, TNF-α, IFNγ, or LPS in adipocytes (61). LPS activates toll-like receptor 4 (TLR4), one of the TLR family of pattern recognition receptors that regulate inflammatory responses in RA. Treatment of mice with LPS induced ANGPTL4 expression in adipose tissue and muscle that was dependent on TLR4 signalling (61, 62). Additionally, circulating levels of ANGPTL4 positively correlate with the inflammatory marker C reactive protein in patients with inflammatory conditions such as metabolic syndrome, type 2 diabetes, and chronic obstructive pulmonary disease (63–65) as well as within the general population (66). These extra-articular conditions often accompany RA and also relate to insulin resistance, leading to suggestions that ANGPTL4 might represent a molecular link between insulin resistance and RA (67).

However, few direct effects of ANGPTL4 on inflammation are yet described. ANGPTL4 protected against the lethal inflammation induced by dietary saturated fat in mice, by reducing inflammatory gene expression and macrophage foam cell formation (68). However, it was shown to expand the proliferation and formation of myeloid progenitors (69). Further studies are required to determine whether ANGPTL4 mediates pro- or anti-inflammatory effects in RA.

## ANGPTL4 in Other Musculoskeletal Conditions

### Cancer in Bone

Angiopoietin-like 4 is overexpressed in the hypoxic peri-necrotic regions of solid tumours and has central roles in cancer growth, anoikis resistance, angiogenesis, and metastasis (54, 55). These pro-tumourigenic mechanisms are covered in other reviews, although pro-metastatic effects generally relate to promotion of angiogenesis and vascular permeability.

There are, however, few descriptions of ANGPTL4 in primary bone tumours or cancer metastasis to bone. Breast cancer, prostate cancer, and lung cancer most commonly metastasise to bone. ANGPTL4 has only been described in relation to breast cancer metastasis, but its pro-metastatic role was largely related to distant lung or brain metastases with little mention of bone metastatic disease.

Angiopoietin-like 4 is part of gene signatures associated with distant metastasis (70) and tumour aggressiveness (71) in breast cancer and is overexpressed in high-grade breast carcinoma (72). However, bone metastatic disease was not apparently included in these analyses. Padua et al. showed ANGPTL4 knock-down to reduce breast cancer lung metastasis, but with no effect on either local lymph node metastasis or bone metastasis (73). Similarly, ANGPTL4 overexpression increased breast cancer lung metastasis by MDA-MB-231 cells (74).

Osteosarcoma, Ewing’s sarcoma, and chondrosarcoma are the most common primary bone tumours, and HIF promotes disease progression in each (75–78), as well as in giant cell tumour of bone (GCTB) (79) and multiple myeloma (80). We detected ANGPTL4 expression in osteoclasts and mononuclear cells present in GCTB (17). Contact of multiple myeloma cells with mesenchymal stem cells or pre-osteoblasts increased expression of ANGPTL4 in the non-malignant population and enhanced myeloma cell adhesion (81). Considering the pro-tumourigenic effects of HIF in these cancers, ANGPTL4 might be expected to also exert tumour-promoting effects, but this has not been investigated.

### Bone Fracture and Osteoporosis

Hopwood et al. compared gene expression in trabecular bone from the proximal femur of osteoporotic (OP) individuals who had suffered a fragility fracture of the femur with bone from age-matched controls (82). Three groups of genes overexpressed in OP were involved in osteoclast differentiation and function, inhibition of osteoblast differentiation and mineralisation, or were genes involved in adipogenesis, lipid metabolism, glucose metabolism, and insulin resistance. ANGPTL4 was one of these genes (82) and, with roles in all three disease processes, could be considered a critical mediator of OP pathology.

Angiopoietin-like 4 overexpression occurred in newly mineralising osteoblasts in a murine model of stabilised femoral fracture (83), and ANGPTL4 mRNA expression was elevated during osteogenic differentiation of MC3T3-E1 and periodontal ligament cells (83, 84). Exogenous ANGPTL4 enhanced osteoblastogenic gene expression in MC3T3-E1 cells but did not affect mineralisation (83). In osteoblastic Saos2 cells, high ANGPTL4 concentrations promoted proliferation but inhibited osteoblastogenesis, whereas lower concentrations promoted osteoblast differentiation (17). Further definition of the effects of ANGPTL4 on osteoblast formation and function is still required.

### Osteoarthritis

Overexpression of ANGPTL4 also occurs in OA. Microarray studies described ANGPTL4 overexpression in cartilage from non-traumatic osteonecrosis of the femoral head, OA (85), and porcine osteochondrosis (86) versus control cartilage, as well as in damaged versus undamaged cartilage from individuals with anteromedial knee OA (87). We observed immunohistochemical overexpression of ANGPTL4 within the OA synovium, although to a lesser extent than in RA. However, secretion of ANGPTL4 was not elevated in non-inflammatory OA serum (32). Coupled with ANGPTL4-mediated induction of MMP expression and cartilage matrix remodelling in chondrocytes (16, 39), this suggests ANGPTL4 as a potential mediator of pathogenic cartilage destruction in OA.

## Summary

Multiple physiological and pathological roles associated with osteolytic disease are ascribed to ANGPTL4 including promotion of osteoclast-mediated bone resorption, cartilage degradation, angiogenesis and vascular permeability, as well as tumour cell growth and metastasis. However research into the musculoskeletal functions of ANGPTL4 is in its infancy, resulting in some controversy or lack of comprehensive research regarding the precise role(s) of ANGPTL4 in different disease processes. This is further complicated by the assignation of alternative cellular functions to the three cleavage products of ANGPTL4. Direct investigation of effects of ANGPTL4 in the varied and different pathologies is yet to be performed and will need to consider the different cleavage products as well as combined effects of modifying multiple disease processes on overall disease activity.

Despite this, ANGPTL4 must be considered an attractive potential therapeutic target, blockade of which might dramatically affect disease progression *via* inhibition of multiple disease processes. HIF-1α itself is considered a good target for treatment of RA. However, advancement of this hypothesis is limited by the lack of drugs that specifically block the HIF pathway, precluding detailed analysis of specific effects of HIF inhibition on RA progression. Neutralising anti-ANGPTL4 antibodies have been developed for use in murine models of disease (88, 89) and, as interest grows in targeting ANGPTL4 therapeutically, humanised neutralising anti-ANGPTL4 antibodies will likely also be developed. With the ability to specifically inhibit ANGPTL4-mediated disease processes, the path is open for new research into emerging effects of this adipokine in osteolytic disease.

## Author Contributions

HK conceived the review, acquired, and critically analysed the literature, and wrote and critically revised the manuscript.

## Conflict of Interest Statement

The author declares that the research was conducted in the absence of any commercial or financial relationships that could be construed as a potential conflict of interest.
